# Supplementary material for: Occurrence and Distribution of Tetrabromobisphenol A and Diversity of Microbial Community Structure in the Sediments of Mangrove
Source: Biology (Basel). 2023 May 22;12(5):757. doi: 10.3390/biology12050757 (PMC10215683; doi:10.3390/biology12050757)
Supplement: Supplementary file 1 [file biology-12-00757-s001.zip › biology-2355367-supplementary.pdf]

Supporting Information

Occurrence and Distribution of Tetrabromobisphenol A and Diversity of Microbial Community Structure in the Sediments of Mangrove

Yongcan Jiang [1,2,3,*], Qiang Wang [4], Yunling Du [1], Dong Yang [1], Jianming Xu [2] and Chongling Yan [3,*]

1 PowerChina Huadong Engineering Corporation Ltd., Hangzhou 311122, China
2 College of Environmental and Resource Sciences, Zhejiang University, Hangzhou 310058, China
3 College of the Environment and Ecology, Xiamen University, Xiamen 361102, China
4 State Key Laboratory of Herbage Improvement and Grassland Agro-Ecosystems, College of Pastoral Agriculture Science and Technology, Lanzhou University, Lanzhou 730000, China
* Correspondence: jiangyc6@zju.edu.cn (Y.J.); ycl@xmu.edu.cn (C.Y.); Tel.: +86-571-8898-2951 (Y.J.); Fax: +86-571-8697-1359 (Y.J.)

Extraction and purification of TBBPA

The TBBPA extracted method according to Liu et al. (2017) with some modification, the extract was by the accelerated solvent extraction (Dionex, ASE 350) with dichloromethane (DCM):Hexane (2:1, v/v), 5.0 g diatomaceous earth on the filter, and them the freeze-dried sample was ground with copper powder (2.0 g) was added. On top of the ground was filled with diatomite earth. The extraction cell was filled with extraction solvent (DCM: Hexane = 2:1, v/v) until the pressure reached 1500 psi (1 psi = 6894.76 Pa) and meanwhile the temperature heated to 100 °C. After 5.0 min heating time and three times static extraction of 10 min at constant pressure and temperature were performed and the extraction was collected in the vial. The final volume of extract was about 90 mL. Subsequently, the extracts were transferred to a pear flask, concentrated to dryness using rotary evaporation, redissolved in 3 mL acetone, and then further cleaned up via solid phase extraction (SPE). For the SPE cleanup, ENVI-Carb SPE cartridges (product information) were sequentially activated with 10.0 mL DCM and 10.0 mL hexane, followed by sample loading onto cartridges and then elusion of the target analyte with 10 mL DCM: hexane (1:1, v/v) into collection vials before analysis on the UPLC/MS/MS using instrumental parameters listed in Tables S1 and S2.

PCR amplification

The PCR reactions were conducted using the following program: 3 min of denaturation at 95 °C, 27 cycles of 30 s at 95 °C, 30 s for annealing at 55 °C, and 45 s for elongation at 72 °C, and a final extension at 72 °C for 10 min. PCR reactions were performed in

triplicate 20 μL mixture containing 4 μL of 5 × FastPfu Buffer, 2 μL of 2.5 mmol L^{-1} dNTPs, 0.8 μL of each primer (0.5 μM), 0.4 μL of FastPfu Polymerase and 10 ng of template NDA. The PCR products were extracted from a 2% agarose gel and further purified using the AxyPrep NDA Gel Extraction Kit (Axygen Biosciences, Union City, CA, USA) and quantified using QuantiFluor™-ST (Promega, USA) according to the manufacturer's protocol. Primers used for amplification of gene sequences provided in Tables S3.

Data processing

Raw FASTQ files were de-multiplexed using an in-house perl script, and then quality-filtered by fastp version 0.19.6[2] and merged by FLASH version 1.2.7[3] with the following criteria:

(i) the 300 bp reads were truncated at any site receiving an average quality score of <20 over a 50 bp sliding window, and the truncated reads shorter than 50 bp were discarded, reads containing ambiguous characters were also discarded; (ii) only overlapping sequences longer than 10 bp were assembled according to their overlapped sequence. The maximum mismatch ratio of overlap region is 0.2. Reads that could not be assembled were discarded; (iii) Samples were distinguished according to the barcode and primers, and the sequence direction was adjusted, exact barcode matching, 2 nucleotide mismatch in primer matching. Then the optimized sequences were clustered into operational taxonomic units (OTUs) using UPARSE 7.1 [4,5] with 97% sequence similarity level. The most abundant sequence for each OTU was selected as a

representative sequence. To minimize the effects of sequencing depth on alpha and beta diversity measure, the number of 16S rRNA gene sequences from each sample were rarefied to 20,000, which still yielded an average Good’s coverage of 99.09%, respectively. The taxonomy of each OTU representative sequence was analyzed by RDP Classifier version 2.2[6] against the 16S rRNA gene database (Silva v138) using confidence threshold of 0.7.

.

Table S1. Gradient injection procedure.

Time (min)	Mobile A (%)	Mobile B (%)
0.00	30.00	70.00
4.00	20.00	80.00
9.00	12.00	88.00
9.10	0.00	100.00
14.00	0.00	100.00
14.10	30.00	70.00
20.00	30.00	70.00

Table S2. UPLC-MS/MS mass spectrometry parameters for TBBPA.

Retention time(min)	Target Substance	Molecular Weight	MRM Quantification	Collision Energy(V)	MRM Qualitative	Collision Energy(V)
18.418	TBBPA	543.9	542.7/79	55	542.7/417.8	55
18.484	^{13}C-TBBPA	555.9	554.8/79	55	554.8/428.7	55

Table S3. Primers used for amplification of gene sequences.

Genes	Primers	Sequences
16S rRNA	338F	5'- ACTCCTACGGGAGGCAGCAG-3'
	806R	5'- GGACTACHVGGGTWTCTAAT-3'

Table S4. Concentrations of TBBPA, total organic carbon (TOC), total nitrogen (TN) and pH in mangrove sediment from Zhangjiang estuary mangrove. Sediment samples from sampling sites 4, 5 and, 6 were used for microbial community analysis.

Sampling sites	1	2	3	4	5	6	7	8	9	10
TBBPA	12.30	8.90	1.30	8.40	12.30	8.95	3.70	5.43	6.28	13.47
TOC (%)	1.618	2.369	1.330	1.472	1.946	2.580	1.385	1.706	1.492	1.178
TN (%)	0.139	0.173	0.118	0.139	0.166	0.163	0.141	0.148	0.140	0.102
pH	6.81	6.70	6.73	6.71	6.77	6.71	6.73	6.70	6.71	6.84
Sampling sites	11	12	13	14	15	16	17	18	19	20
TBBPA	7.60	10.50	1.80	9.14	20.46	10.48	8.88	4.83	5.48	7.85
TOC (%)	2.015	2.286	2.882	2.328	2.943	1.439	1.817	1.856	2.109	2.542
TN (%)	0.146	0.154	0.214	0.183	0.246	0.118	0.129	0.147	0.159	0.175
pH	6.72	6.73	6.70	6.65	6.66	6.63	6.64	6.49	6.73	6.51
Sampling sites	21									
TBBPA	7.00									
TOC (%)	2.363									
TN (%)	0.129									
pH	6.62									

Table S5. Concentrations of TBBPA, total organic carbon (TOC), total nitrogen (TN) and pH in mangrove sediment from Jiulongjiang estuary mangrove. Sediment samples from sampling sites 4, 5 and, 6 were used for microbial community analysis.

Sites	1	2	3	4	5	6	7	8	9	10
TBBPA	40.77	20.78	40.58	18.04	12.03	9.18	9.30	13.86	8.33	8.16
TOC (%)	1.441	1.728	1.386	1.554	1.789	1.375	1.860	1.306	2.392	1.558
TN (%)	0.174	0.117	0.138	0.175	0.119	0.138	0.139	0.122	0.113	0.101
pH	6.78	6.80	6.77	6.64	6.65	6.77	6.89	6.83	6.41	6.74
Sites	11	12	13	14	15	16	17	18	19	20
TBBPA	9.08	9.35	6.01	7.08	6.67	8.17	7.54	9.13	8.54	7.58
TOC (%)	2.273	1.390	1.654	2.119	1.554	1.688	1.530	1.269	1.181	1.024
TN (%)	0.133	0.108	0.116	0.143	0.137	0.136	0.140	0.145	0.158	0.124
pH	6.48	6.68	6.66	6.64	6.38	6.81	6.77	6.6	6.72	6.69
Sites	21	22	23	24	25	26				
TBBPA	7.55	12.10	5.54	6.17	3.47	7.45				
TOC (%)	1.481	1.235	1.269	1.716	1.475	2.289				
TN (%)	0.125	0.150	0.126	0.135	0.115	0.146				
pH	6.43	6.45	6.42	6.44	6.69	6.64				

Table S6. Concentrations of TBBPA, total organic carbon (TOC), total nitrogen (TN) and pH in mangrove sediment from Quanzhou bay estuary mangrove. Sediment samples from sampling sites 1, 2 and, 3 were used for microbial community analysis.

Sites	1	2	3	4	5	6	7	8	9	10
TBBPA	19.83	11.36	8.79	10.71	7.91	2.37	15.97	7.14	8.94	10.94
TOC (%)	1.060	1.154	1.106	1.037	1.085	1.123	1.167	1.111	1.432	1.076
TN (%)	0.099	0.095	0.091	0.095	0.093	0.129	0.102	0.116	0.129	0.096
pH	6.76	6.79	6.85	6.67	6.62	6.51	6.78	6.43	6.73	6.72
Sites	11	12	13	14	15	16	17	18	19	20
TBBPA	8.80	12.23	9.33	7.24	11.21	7.53	8.48	18.36	7.53	5.87
TOC (%)	1.083	1.160	1.034	1.017	1.088	2.169	2.000	1.630	1.374	1.539
TN (%)	0.098	0.115	0.100	0.097	0.089	0.130	0.106	0.132	0.123	0.119
pH	6.80	6.67	6.58	6.50	6.81	6.75	6.47	6.48	6.57	6.62
Sites	21	22	23							
TBBPA	10.14	7.72	6.49							
TOC (%)	1.037	1.492	1.731							
TN (%)	0.097	0.131	0.145							
pH	6.56	6.68	6.64							

Table S7. Summary of 16S rRNA Illumina MiSeq Paired-End 300 sequences, operational taxonomic units (OTUs) and phylotype coverages information for Zhangjiang estuary, Jiulongjiang estuary, and Quanzhou bay estuary mangrove sediments.

Locations	Phylum	Class	Order	Family	Genus	Species	OTU
All	61	158	303	544	1052	22998	9436
ZJ	60	148	279	498	940	1970	7337
JLJ	58	149	276	496	948	1999	7601
QZ	58	151	293	509	962	2057	7900

Table S8. Microbial α-diversity indices of Zhangjiang estuary mangrove, Jiulongjiang estuary mangrove, and Quanzhou bay estuary mangrove sediments.

Locations	Shannon	Sobs	Ace	Chao 1	Coverage
ZJ	7.02	5072.33	7036.53	6971.06	0.967
JLJ	7.16	5608.67	7862.49	7701.81	0.9628
QZ	7.22	5529	7603.12	7507.88	0.9650

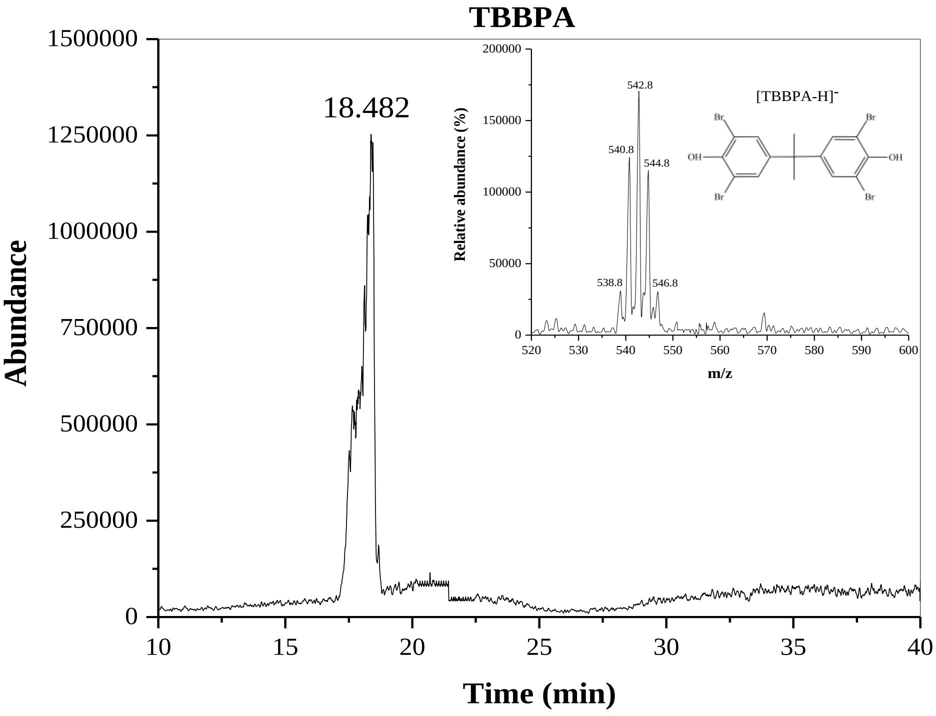

Figure S1. Chromatogram of TBBPA detected by high performance liquid chromatography tandem mass spectrometry.

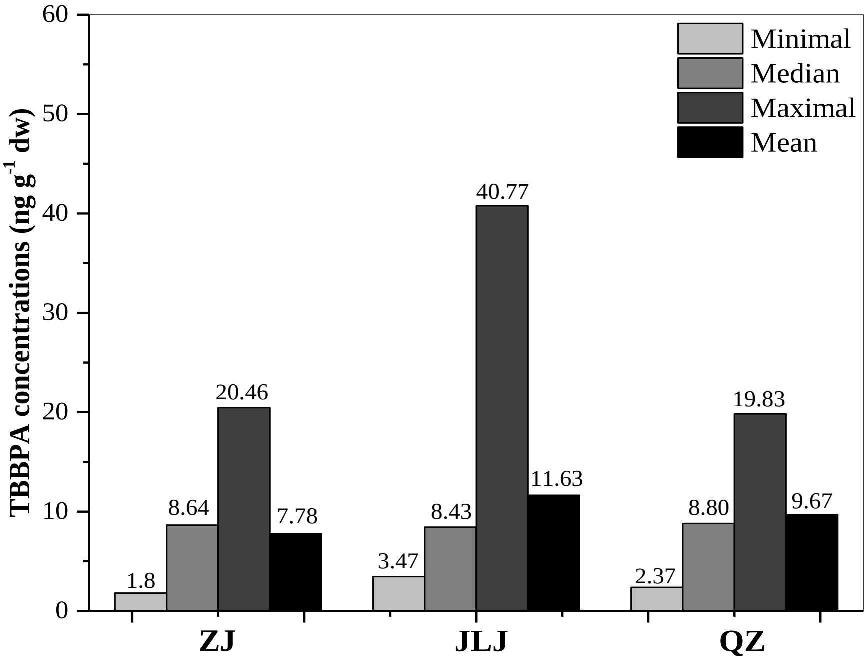

Figure S2. Minimum, median, maximum and average TBBPA concentrations in ZJ, JLJ and QZ sediment. ZJ, JLJ, and QZ indicate Zhangjiang estuary mangrove sediment, Jiulongjiang estuary mangrove sediment and Quanzhou bay estuary mangrove sediment, respectively.

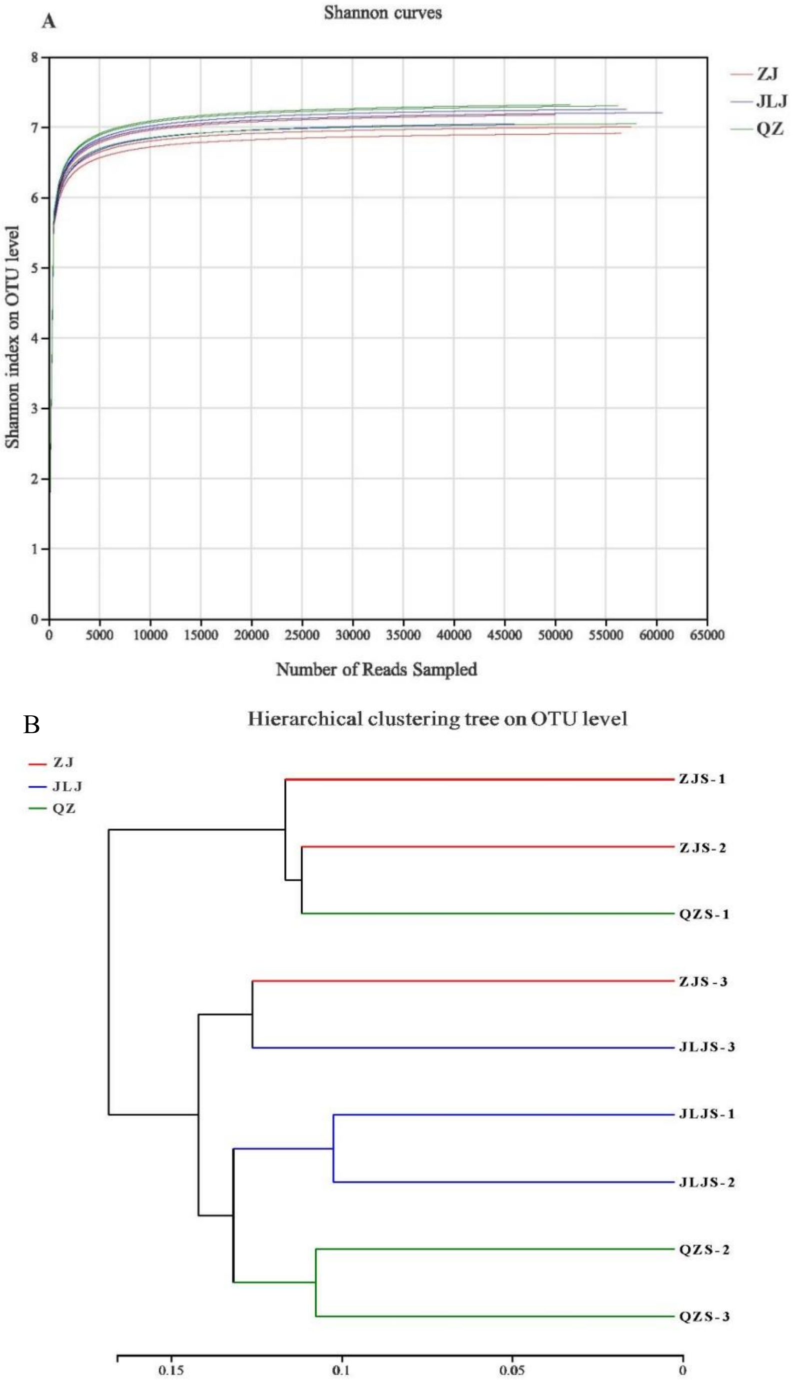

Figure S3. (A) Rarefaction of Shannon index of OTU level in all sediment samples. (B) The OUT level-based hierarchical cluster showing the difference of bacterial community between ZJ sediment, JLJ sediment, and QZ sediment. ZJ, JLJ, and QZ indicate Zhangjiang estuary mangrove sediment, Jiulongjiang estuary mangrove sediment and Quanzhou bay estuary mangrove sediment, respectively.

Community analysis on Phylum level : ZJ

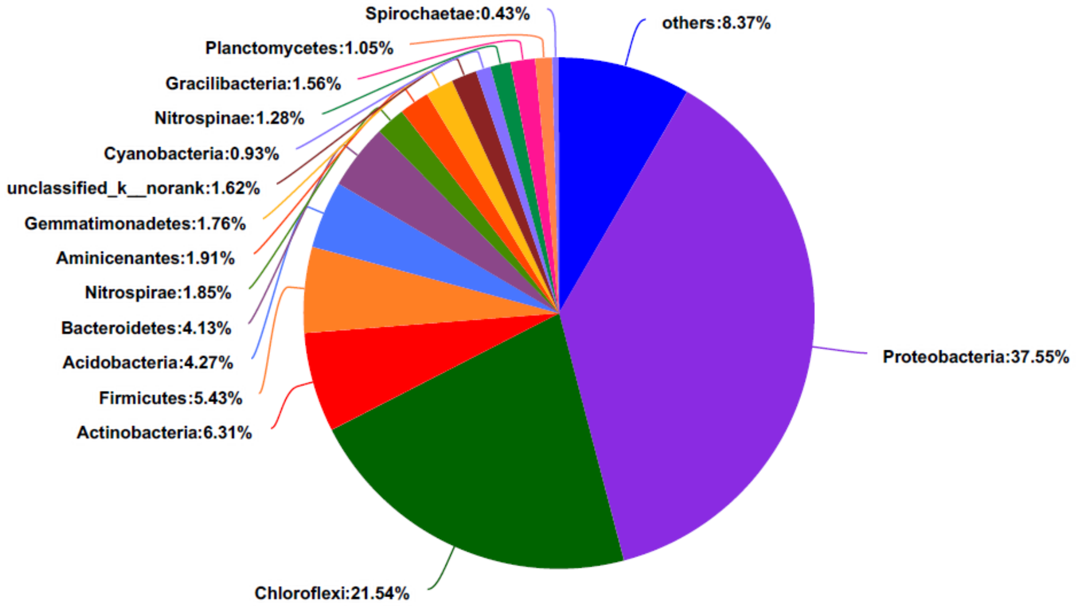

Community analysis on Phylum level: JLJ

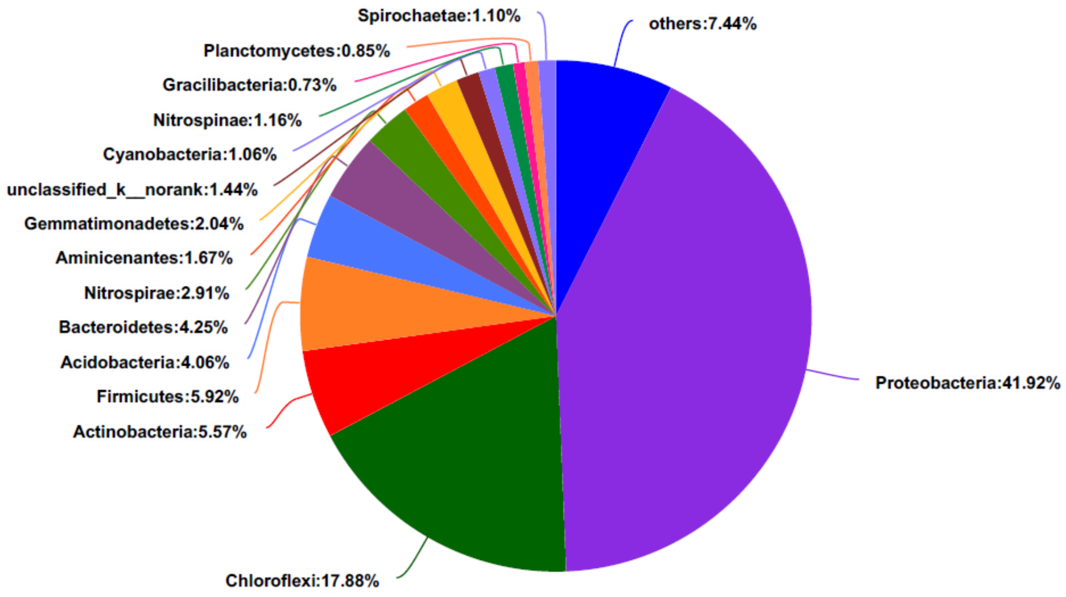

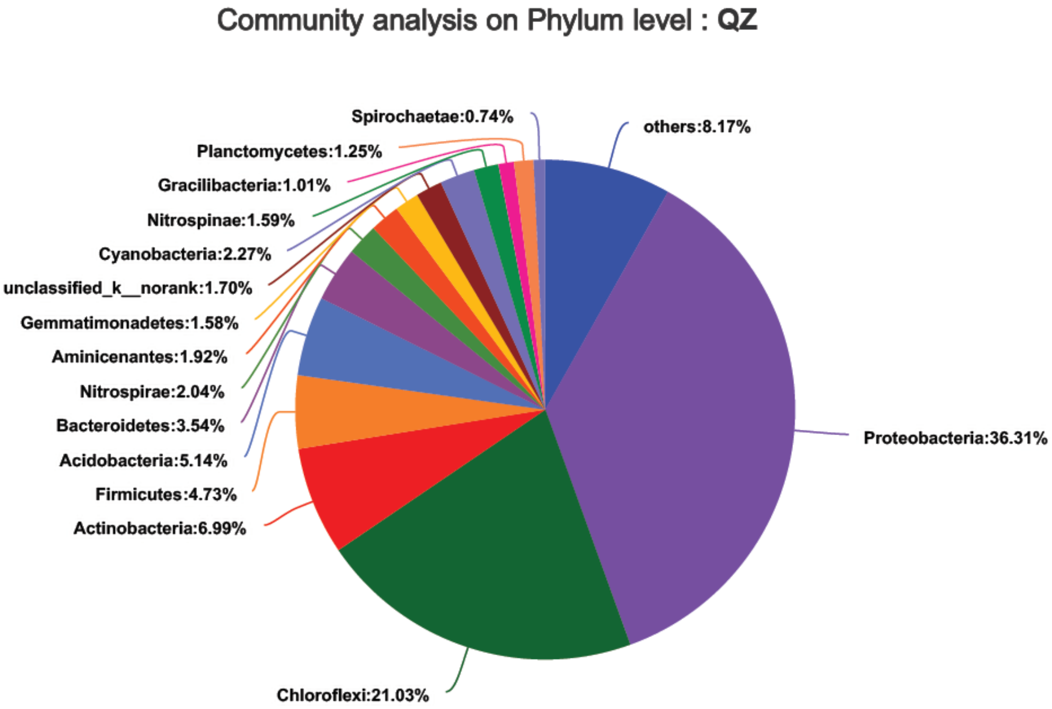

Figure S4. Dominant microbial community structure composition at phylum level in the Zhangjiang estuary mangrove sediment (ZJ), Jiulongjiang estuary mangrove sediment (JLJ) and Quanzhou bay estuary mangrove sediment (QZ).

Community analysis on Genus level : ZJ

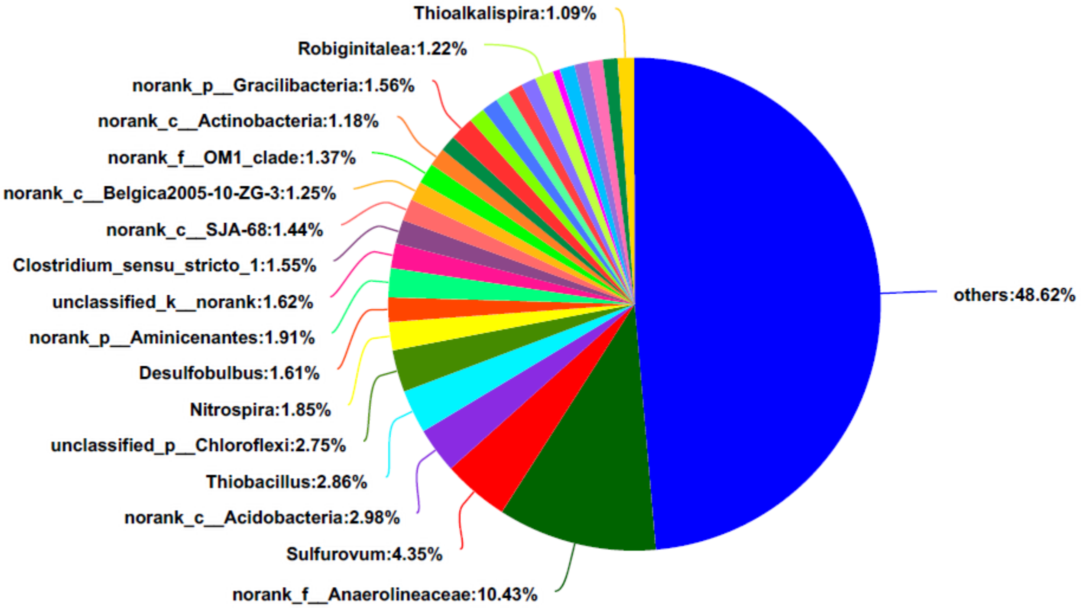

Community analysis on Genus level : JLJ

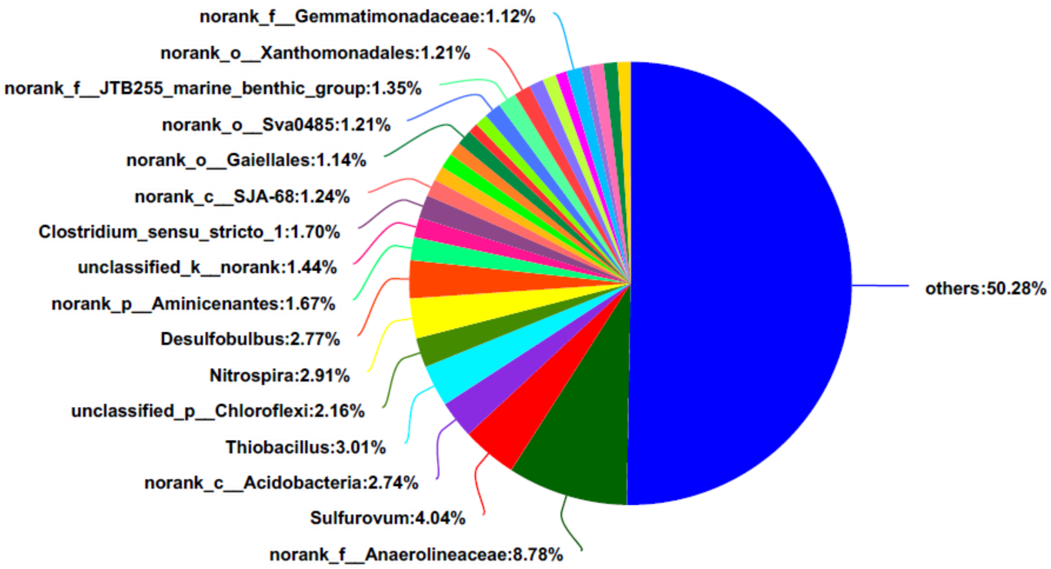

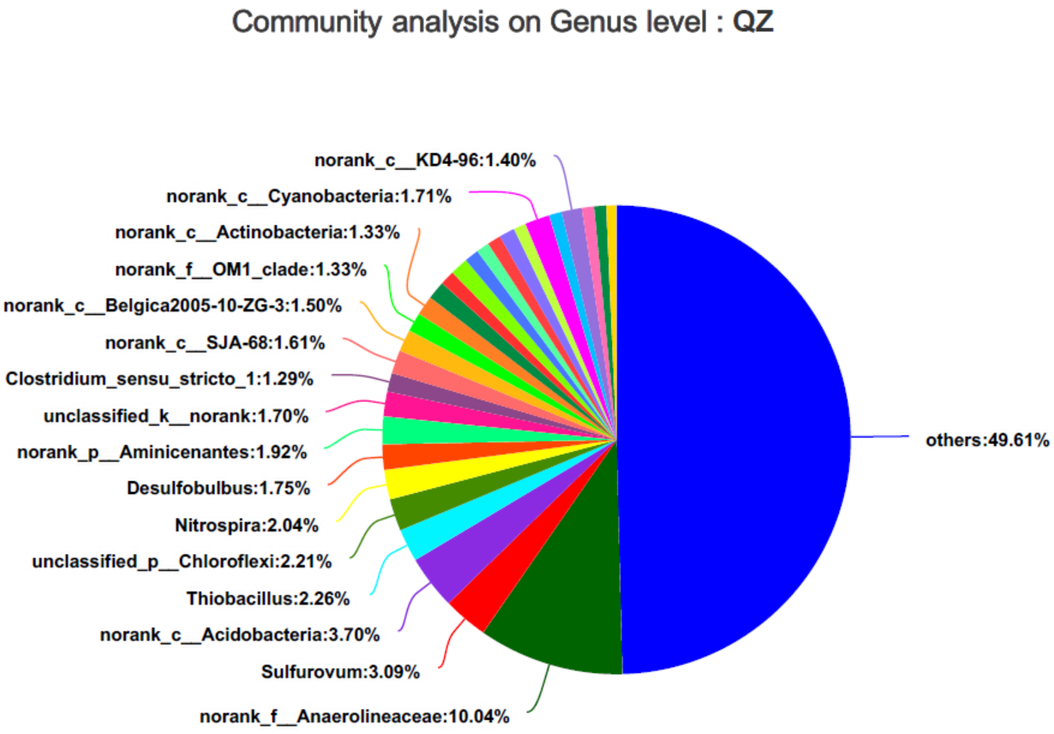

Figure S5. Dominant microbial community structure composition at gunes level in the Zhangjiang estuary mangrove sediment (ZJ), Jiulongjiang estuary mangrove sediment (JLJ) and Quanzhou bay estuary mangrove sediment (QZ).

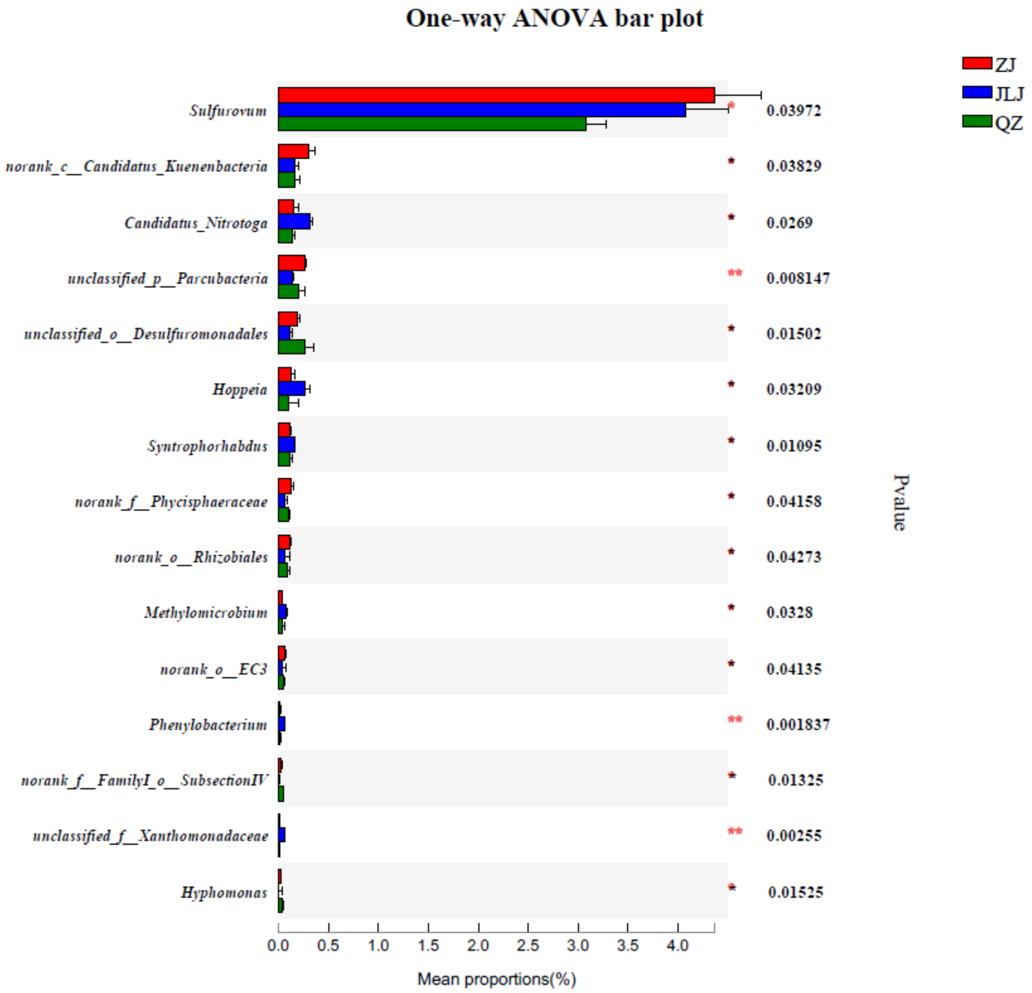

Figure S6. Significant test analysis of the top 15 genus between three mangrove sediments: Zhangjiang estuary mangrove sediment (ZJ), Jiulongjiang estuary mangrove sediment (JLJ) and Quanzhou bay estuary mangrove sediment (QZ) (one-way ANOVA, *$P < 0.05$, **$P < 0.01$, ***$P < 0.001$).

Reference:

[1] Liu, A., Shi, J., Qu, G., Hu, L., Ma, Q., Song, M., Jing, C., Jiang, G., 2017. Identification of Emerging Brominated Chemicals as the Transformation Products of Tetrabromobisphenol A (TBBPA) Derivatives in Soil. Environmental Science & Technology 51, 5434-5444.

[2] Chen S , Zhou Y , Chen Y , et al. fastp: an ultra-fast all-in-one FASTQ preprocessor[J]. Bioinformatics, 2018, 34(17)

[3] Tanja, Mago, Steven, et al. FLASH: fast length adjustment of short reads to improve genome assemblies.[J]. Bioinformatics, 2011, 27(21):2957-2963.

[4] Edgar, Robert C . UPARSE: highly accurate OTU sequences from microbial amplicon reads.[J]. Nature Methods, 2013, 10(10):996-998.

[5] Stackebrandt E , Goebel B M . Taxonomic Note: A Place for DNA-DNA Reassociation and 16S rRNA Sequence Analysis in the Present Species Definition in Bacteriology[J]. Int.j.syst.bacteriol, 1994, 44(4):846-849.

[6] Wang Q . Naive Bayesian classifier for rapid assignment of rRNA sequences into the new bacterial taxonomy[J]. Appl. Environ. Microbiol, 2007, 73.
